# Health Care Use and Costs of Children, Adolescents, and Young Adults With Somatic Symptom and Related Disorders

**DOI:** 10.1001/jamanetworkopen.2020.11295

**Published:** 2020-07-23

**Authors:** Natasha Ruth Saunders, Sima Gandhi, Simon Chen, Simone Vigod, Kinwah Fung, Claire De Souza, Hana Saab, Paul Kurdyak

**Affiliations:** 1 The Hospital for Sick Children, Toronto, Ontario, Canada; 2 Department of Pediatrics, University of Toronto, Toronto, Ontario, Canada; 3 ICES, Toronto, Ontario, Canada; 4 Child Health Evaluative Sciences, SickKids Research Institute, Toronto, Ontario, Canada; 5 Institute of Health Policy, Management and Evaluation, The University of Toronto, Toronto, Ontario, Canada; 6 Women’s College Research Institute, Women’s College Hospital, Toronto, Ontario, Canada; 7 Department of Psychiatry, University of Toronto, Toronto, Ontario, Canada; 8 Centre for Addiction and Mental Health, Toronto, Ontario, Canada

## Abstract

**Importance:**

Somatic symptom and related disorders are highly prevalent mental health disorders among young people. Presentation can be varied, and patients often face long delays and see multiple practitioners to receive a diagnosis.

**Objective:**

To evaluate the health care use and costs in a population-based sample of children and young people with somatic symptom and related disorders in Ontario, Canada.

**Design, Setting, and Participants:**

This population-based cohort study used linked health and administrative databases in Ontario, Canada, where health services are funded through a universal single-payer health insurance plan. Participants included children aged 4 to 12 years, adolescents aged 13 to 17 years, and young adults aged 18 to 24 years residing in Ontario, Canada, during the period of April 1, 2008, to March 31, 2015. Included participants had a first health record diagnosis of somatic symptom and related disorders and were grouped based on the setting of their index somatic symptom and related disorders contact: outpatient, emergency department, or inpatient. Data were analyzed from August 1, 2017, to February 1, 2018.

**Exposures:**

One year before and 1 year after diagnosis of somatic symptom and related disorders.

**Main Outcomes and Measures:**

Outcome measures included overall and mental health–specific ambulatory and acute care visits and overall health system costs and sector-specific costs.

**Results:**

A total of 33 272 patients (median [interquartile range {IQR}] age, 20 [16-22] years; 17 387 female [52.3%]) were included in the analysis. Among these patients, 3875 (11.6%) were aged 4 to 12 years, 7273 (21.9%) were aged 13 to 17 years, and 22 124 (66.5%) were aged 18 to 24 years. A total of 17 893 (53.8%) had their index visit as outpatients, whereas 13 310 (40.0%) and 2069 (6.2%) were diagnosed in the emergency department and inpatient settings, respectively. Ambulatory physician visits were frequent and persisted 1 year after diagnosis within each setting (before vs after 1 year, median [IQR] visits, inpatient setting: 7 [3-13] vs 7 [3-13]; emergency department setting: 4 [2-8] vs 4 [2-9]; outpatient setting: 3 [1-7] vs 4 [2-7]; *P* < .001). After diagnosis, many did not receive physician-delivered mental health care (35.3% [730 of 2069] in an inpatient setting, 59.1% [7866 of 13 310] in an emergency department setting, 58.5% [10 467 of 17 893] in an outpatient setting; *P* < .001). Acute care use was frequent and remained so after diagnosis across settings. Of those hospitalized as inpatients at diagnosis, 37.7% (779 of 2069) were readmitted within 1 year. Mean (SD) 2-year patient costs were CAD$9845 ($39 725) (median [IQR], $2401 [$960-$7019]). Hospitalized patients had a 2-year mean (SD) cost of $51 424 ($100 416) (median [IQR], $21 997 [$12 510-$45 841]) per-patient expenditure.

**Conclusion and Relevance:**

This study found that children and young people with somatic symptom and related disorders frequently used the health system with substantial health system costs before and after diagnosis. Many of these patients did not receive physician-delivered mental health care. These findings suggest that this population may be under-recognized, and initiatives for early recognition and engagement with mental health support may be warranted.

## Key Points

**Question:**

How do children, adolescents, and young adults with somatic symptom and related disorders use the health care system, and what are the direct health system costs associated with this population?

**Findings:**

Results from this population-based cohort study suggest that these individuals had substantial health system use and costs before receipt of a diagnosis, especially for those hospitalized. Frequent use and costs persisted after initial diagnosis, and follow-up care by physicians for mental health was poor.

**Meaning:**

These findings suggest that this population may be under-recognized with inadequately addressed needs. Initiatives for early recognition and engagement with mental health support are warranted.

## Teaser

This cohort study uses data from linked health and administrative databases in Ontario, Canada, to investigate health care use and costs among children, adolescents, and young adults diagnosed with somatic symptom and related disorders.

## Introduction

Somatic symptom and related disorders (SSRDs) are highly prevalent and account for a large proportion of health system visits.^1,2,3,4,5^ Presentation of these psychiatric disorders can be varied, but the core features are physical symptoms inconsistent with history, physical examination, or laboratory or imaging investigations.^2,4,6,7^ Types of SSRDs include somatic symptom disorder, conversion disorder (also called functional neurological symptom disorder), illness anxiety disorder, and psychological factors affecting medical conditions.^8^ In these disorders, psychological or emotional distress is experienced through physical symptoms, known as somatic symptoms. Associated terms include *functional disorders* and, historically, *medically unexplained symptoms*.^4,9^ There is overlap in the diagnostic classification of SSRDs with other conditions, such as fibromyalgia, irritable bowel syndrome, and chronic fatigue syndrome.^10,11,12^ SSRDs are associated with considerable impairment in functioning, including school absenteeism, social isolation, reduced quality of life, unnecessary and sometimes harmful diagnostic testing, and delays in appropriate treatment and intervention for what is actually a primary mental health disorder.^1,2,13^ Delays in diagnosis and treatment often lead to frustration and worry in patients and families who may feel dismissed.^1^ Similarly, health care professionals experience distress in failing to improve their patients’ functioning.^13^

An SSRD contributes to substantial personal, health system, and societal costs.^9,14,15,16^ Some work has been done in adults to better characterize health system use and costs in these populations, but little has been done in children, adolescents, and young adults.^9,14,17,18^ Although most patients with somatic symptoms are seen in primary care settings,^13,17^ a proportion of individuals may be so distressed or impaired by their symptoms that they seek care in an emergency department (ED) or are hospitalized (becoming inpatients). In these latter settings, patients may present with a more severe form of the disorder, with high intensity and often unmet health care needs.^1,2,19^ Identification or diagnosis of the disorder, regardless of setting, represents an opportunity for intervention, but acceptance of diagnosis and navigation of treatment pathways are often not easy.^2^ Given the barriers to appropriate care that these patients face, characterizing the patterns of health care use, including the setting in which patients receive their diagnosis and the care received after somatic symptoms have been identified along with associated costs, will shed light on the magnitude of the issue and opportunities for intervention. Our objectives were to describe the sociodemographic and clinical characteristics of children, adolescents, and young adults with an SSRD in Ontario, Canada’s largest province with a population of almost 14 million, and to describe the patterns of health care use and costs in the population in the year leading up to and the year after diagnosis.

## Methods

### Study Design

This population-based cohort study used linked health and demographic administrative databases from Ontario and housed at ICES, a not-for-profit research institute whose legal status under Ontario’s health information privacy law allows it to collect and analyze health data without consent. Databases are linked through a uniquely encoded health identification number derived from the health card number of every resident in Ontario with provincial health insurance. Research ethics board approval for this study was received from The Hospital for Sick Children. This study followed the Strengthening the Reporting of Observational Studies in Epidemiology (STROBE) reporting guideline.

### Data Sources

The study cohort was constructed using diagnostic codes from provincial portions of hospital discharge (Discharge Abstract Database, Ontario Mental Health Reporting System), ED and same-day surgery (National Ambulatory Care Reporting System), and provincial physician billing (Ontario Health Insurance Plan) data sets. The Registered Persons Database, Ontario’s health care registry, contains demographic and vital statistics data for all Ontario residents eligible for public health insurance and was used for capturing demographic data. The Ontario portion of Immigration, Refugees, and Citizenship Canada’s Permanent Resident Database was used for immigration information. A complete list of databases and variables used are available in eTable 1 in the Supplement.

### Study Population

All children, adolescents, and young adults aged 4 to 24 years living in Ontario between April 1, 2008, and March 31, 2015, were included. A cutoff age of 24 years was used, as this is the age cutoff used to define youth by the United Nations.^20^ This age group was further stratified as children (4-12 years), adolescents (13-17 years), and young adults (18-24 years). The study cohort comprised any individual who was seen as an outpatient and had a physician billing code for psychosomatic disturbances (Ontario Health Insurance Plan diagnostic code 306) or any individual who was discharged from a hospital or ED with a discharge diagnosis that included any of the following: somatization disorder, conversion disorder, factitious disorder, irritable bowel syndrome, fibromyalgia, chronic fatigue syndrome, or other related disorders using the *International Statistical Classification of Diseases and Related Health Problems, Tenth Revision–Canada (ICD-10-CA)* diagnostic codes (eTable 2 in the Supplement). No validated definition of an SSRD using health and administrative data from *ICD-10-CA* codes exists, nor is there consensus on clinical diagnoses. However, these health care professional–assigned diagnoses from clinical encounters were chosen because they are the most commonly used for research in SSRDs.^21,22,23^ The first date on which individuals had their SSRD diagnosis in any of the outpatient or acute care databases was considered the index date. A 2-year lookback was used to ascertain that the visit was the first time somatic symptoms were documented and to identify the clinician who provided the majority of primary care. We categorized continuity of primary care in the 2 years leading up to the index SSRD visit as low, moderate, and high with cutoffs of less than 50%, 50% to 79.9%, and 80% or more of all primary care visits to their assigned primary care professional.

### Health Services Utilization

Each individual’s health system use and resulting costs were examined in the 1-year period immediately before diagnosis, including the setting where the index diagnosis was made, and in the 1-year period immediately after this contact. Health system use included (1) all-cause and mental health–related ED visits and hospitalizations and (2) all-cause and mental health–related outpatient visits to family physicians, pediatricians, and psychiatrists, stratified by location of index diagnosis (eg, outpatient, ED, or inpatient setting). Mental health visits included any type of mental illness, not only SSRDs, and were determined based on whether the primary discharge diagnosis code was any code from the chapter “Mental and Behavioral Disorders” in the *ICD-10-CA* (codes F04 to F99). Where Ontario Health Insurance Plan diagnosis codes were used for outpatient visits, a previously validated algorithm for ambulatory mental health care using administrative data was applied.^24^ All visits to psychiatrists were considered mental health related.

Health care costs were based on unit costs of services provided to patients during an episode of care, paid by the Ontario Health Insurance Plan to eligible health care professionals. Acute hospitalizations and ED visit costs were calculated using case-mix methodology, in which the cost of a patient encounter is based on the intensity of resources used during the episode of care. Psychiatric hospitalization costs were determined using measures of resource intensity, days of stay, and case-mix index. Additional information on case-mix costing methodology in Ontario is available and published elsewhere.^25^

### Statistical Analysis

Baseline characteristics were reported as numbers and proportions, stratified by health care setting of index diagnosis and by clinically relevant age groups (school-aged children [4-12 years], adolescents [13-17 years], young adults [18-24 years]). The numbers, proportions, and median visits per patient were compared in the year before and the year after the index diagnosis within each diagnostic setting. Costs were estimated and compared in the year before and the year after diagnosis using means and medians adjusted to the 2016 Canadian dollar. Mean numbers of visits and costs were tested using paired *t* tests, and the McNemar test was used for testing proportions. *P* values were 2-sided, and *P* < .05 was used as the set point for statistical significance. All statistical analyses were conducted from August 1, 2017, to February 1, 2018, using SAS Enterprise Guide, version 6.1 (SAS Institute).

## Results

A total of 33 272 children, adolescents, and young adults (median [interquartile range {IQR}] age, 20 [16-22] years; 17 387 female [52.3%] and 15 885 male [47.7%]) with an SSRD diagnosis between April 1, 2008, and March 31, 2015, were included in the analysis. Among these patients, 3875 (11.6%) were aged 4 to 12 years, 7273 (21.9%) were aged 13 to 17 years, and 22 124 (66.5%) were aged 18 to 24 years. A total of 17 893 (53.8%) had their index visit as outpatients, whereas 13 310 (40.0%) and 2069 (6.2%) were diagnosed in ED and inpatient settings, respectively (Table 1). The majority of those diagnosed while hospitalized were female (1448 of 2069 [70.0%]), whereas the majority of those diagnosed in an outpatient setting were male (10 745 of 17 893 [60.1%]). This trend was consistent across age ranges except in adolescents; among those diagnosed in outpatient settings, 1788 of 3387 were female (52.8%). Individuals were equally distributed across neighborhood income quintiles. Most (30 515 of 33 272 individuals [91.7%]) had a primary care practitioner with whom they followed up regularly, though 11 933 of 33 272 patients (35.9%) had low continuity of primary care (Table 1).

**Table 1.** Baseline Characteristics of Children, Adolescents, and Young Adults With a New Health Record of Somatic Symptom and Related Disorders^a^

Variable	Diagnostic setting^b^												
	Overall (n = 33 272)	4-24 y			4-12 y			13-17 y			18-24 y		
		Outpatient (n = 17 893)	ED (n = 13 310)	Inpatient (n = 2069)	Outpatient (n = 1958)	ED (n = 1608)	Inpatient (n = 309)	Outpatient (n = 3387)	ED (n = 3142)	Inpatient (n = 744)	Outpatient (n = 12 548)	ED (n = 8560)	Inpatient (n = 1016)
Age, median (IQR), y	20 (16-22)	20 (17-22)	19 (16-22)	17 (15-21)	9 (6-11)	9 (7-11)	10 (9-12)	16 (14-17)	16 (14-17)	16 (14-17)	21 (20-23)	21 (19-23)	21 (19-23)
Age, y													
4-12	3875 (11.6)	1958 (10.9)	1608 (12.0)	309 (14.9)	NA	NA	NA	NA	NA	NA	NA	NA	NA
13-17	7273 (21.9)	3387 (18.9)	3142 (23.6)	744 (36.0)	NA	NA	NA	NA	NA	NA	NA	NA	NA
18-24	22 124 (66.5)	12 548 (70.1)	8560 (64.3)	1016 (49.1)	NA	NA	NA	NA	NA	NA	NA	NA	NA
Sex													
Female	17 387 (52.3)	7148 (39.9)	8791 (66.0)	1448 (70.0)	905 (46.2)	839 (52.2)	187 (60.5)	1788 (52.8)	2240 (71.3)	560 (75.3)	4455 (35.5)	5712 (66.7)	701 (69.0)
Male	15 885 (47.7)	10 745 (60.1)	4519 (34.0)	621 (30.0)	1053 (53.8)	769 (47.8)	122 (39.5)	1599 (47.2)	902 (28.7)	184 (24.7)	8093 (64.5)	2848 (33.3)	315 (31.0)
Neighborhood income quintile													
1 (lowest)	7477 (22.5)	3751 (21.0)	3281 (24.7)	445 (21.5)	519 (26.5)	372 (23.1)	56 (18.1)	695 (20.5)	677 (21.5)	133 (17.9)	2537 (20.2)	2232 (26.1)	259 (25.5)
2	6367 (19.1)	3336 (18.6)	2643 (19.9)	388 (18.8)	394 (20.1)	296 (18.4)	54 (17.5)	610 (18.0)	603 (19.2)	145 (19.5)	2332 (18.6)	1744 (20.4)	189 (18.6)
3	6241 (18.8)	3344 (18.7)	2497 (18.8)	400 (19.3)	332 (17.0)	302 (18.8)	76 (24.6)	648 (19.1)	600 (19.1)	139 (18.7)	2364 (18.8)	1595 (18.6)	185 (18.2)
4	6685 (20.1)	3629 (20.3)	2639 (19.8)	417 (20.2)	368 (18.8)	333 (20.7)	56 (18.1)	711 (21.0)	677 (21.5)	153 (20.6)	2550 (20.3)	1629 (19.0)	208 (20.5)
5 (highest)	6383 (19.2)	3784 (21.1)	2189 (16.4)	410 (19.8)	339 (17.3)	293 (18.2)	67 (21.7)	714 (21.1)	570 (18.1)	168 (22.6)	2731 (21.8)	1326 (15.5)	175 (17.2)
Missing	119 (0.4)	49 (0.3)	61 (0.5)	9 (0.4)	6 (0.3)	12 (0.7)	NA	9 (0.3)	15 (0.5)	6 (0.8)	34 (0.3)	34 (0.4)	NA
Rurality													
Major urban	23 449 (70.5)	14 490 (81.0)	7554 (56.8)	1405 (67.9)	1652 (84.4)	935 (58.1)	211 (68.3)	2722 (80.4)	1627 (51.8)	500 (67.2)	10 116 (80.6)	4992 (58.3)	694 (68.3)
Urban	6876 (20.7)	2569 (14.4)	3835 (28.8)	472 (22.8)	217 (11.1)	436 (27.1)	70 (22.7)	475 (14.0)	978 (31.1)	170 (22.8)	1877 (15.0)	2421 (28.3)	232 (22.8)
Rural	2652 (8.0)	780 (4.4)	1712 (12.9)	160 (7.7)	83 (4.2)	206 (12.8)	28 (9.1)	179 (5.3)	479 (15.2)	60 (8.1)	518 (4.1)	1027 (12.0)	76 (7.5)
Missing	295 (0.9)	54 (0.3)	209 (1.6)	32 (1.5)	6 (0.3)	31 (1.9)	NA	11 (0.3)	58 (1.8)	14 (1.9)	37 (0.3)	120 (1.4)	14 (1.4)
Immigrant category													
Refugee	908 (2.7)	673 (3.8)	194 (1.5)	41 (2.0)	38 (1.9)	15 (0.9)	2 (0.6)	114 (3.4)	25 (0.8)	15 (2.0)	521 (4.2)	154 (1.8)	24 (2.4)
Immigrant	2711 (8.1)	2101 (11.7)	521 (3.9)	89 (4.3)	146 (7.5)	39 (2.4)	11 (3.6)	287 (8.5)	89 (2.8)	21 (2.8)	1668 (13.3)	393 (4.6)	57 (5.6)
Nonimmigrant	29 653 (89.1)	15 119 (84.5)	12 595 (94.6)	1939 (93.7)	1774 (90.6)	1554 (96.6)	296 (95.8)	2986 (88.2)	3028 (96.4)	708 (95.2)	10 359 (82.6)	8013 (93.6)	935 (92.0)
Usual practitioner													
Pediatrician	648 (1.9)	270 (1.5)	276 (2.1)	102 (4.9)	110 (5.6)	127 (7.9)	40 (12.9)	98 (2.9)	110 (3.5)	52 (7.0)	62 (0.5)	39 (0.5)	10 (1.0)
Family physician	30 515 (91.7)	16 631 (92.9)	12 038 (90.4)	1846 (89.2)	1745 (89.1)	1321 (82.2)	244 (79.0)	3111 (91.9)	2782 (88.5)	641 (86.2)	11 775 (93.8)	7935 (92.7)	961 (94.6)
Other	87 (0.3)	41 (0.2)	30 (0.2)	16 (0.8)	8 (0.4)	9 (0.6)	NA	8 (0.2)	9 (0.3)	7 (0.9)	25 (0.2)	12 (0.1)	NA
No primary care	2022 (6.1)	951 (5.3)	966 (7.3)	105 (5.1)	95 (4.9)	151 (9.4)	25 (7.1)	170 (5.0)	241 (7.7)	44 (5.9)	686 (5.5)	574 (6.7)	44 (4.4)
Continuity of primary care													
Low	11 933 (35.9)	6689 (37.4)	4535 (34.1)	709 (34.3)	646 (33.0)	502 (31.2)	104 (33.7)	1095 (32.3)	900 (28.6)	242 (32.5)	4948 (39.4)	3133 (36.6)	363 (35.7)
Moderate	8985 (27.0)	4851 (27.1)	3534 (26.6)	600 (29.0)	590 (30.1)	417 (25.9)	101 (32.7)	945 (27.9)	852 (27.1)	213 (28.6)	3316 (26.4)	2265 (26.5)	286 (28.1)
High	10 332 (31.1)	5402 (30.2)	4275 (32.1)	655 (31.7)	627 (32.0)	538 (33.5)	84 (27.2)	1177 (34.8)	1149 (36.6)	245 (32.9)	3598 (28.7)	2588 (30.2)	326 (32.1)
No primary care	2022 (6.1)	951 (5.3)	966 (7.3)	105 (5.1)	95 (4.9)	151 (9.4)	20 (6.5)	170 (5.0)	241 (7.7)	44 (5.9)	686 (5.5)	574 (6.7)	41 (4.0)

Abbreviations: ED, emergency department; IQR, interquartile range; NA, not available.

^a^ In Ontario, Canada, from 2010 to 2015.

^b^ Data are presented as No. (%) unless otherwise specified.

Of the 13 310 patients diagnosed in the ED setting, 7671 (57.6%) had their primary diagnosis as an SSRD, a proportion consistent across all age groups. Of the 2069 patients diagnosed during a hospitalization, 777 (37.6%) had an SSRD as the primary diagnosis, with this proportion reaching 151 of 309 (48.9%) in school-aged children (eTable 3 in the Supplement). Where SSRD was listed as a comorbid diagnosis, the primary diagnosis contributing to the hospitalization or visit was most often another functional disorder or nonspecific physical symptom, such as unspecified abdominal pain or other mental health disorders (eTable 3 in the Supplement). Outpatient physician billing data only allow for a single diagnosis per visit, and therefore all outpatient index visits had a type of somatic disorder as a primary diagnosis.

### Health System Utilization

#### Acute Care Before and After Diagnosis

Children, adolescents, and young adults first diagnosed with an SSRD during a hospitalization had a mean (SD) of 3.1 (4.4) ED visits (median [IQR], 2 [1-4]) and a mean (SD) of 2.7 (4.9) ED visits (median [IQR], 1 [0-3]) in the year before and the year after diagnosis, respectively (*P* < .001). In the year after index diagnosis, 779 of 2069 patients (37.7%) were hospitalized again, a proportion similar to the year preceding diagnosis (Table 2 and Table 3). Mean (SD) length of stay on the index hospitalization was 6.9 (11.9) days for children aged 4 to 12 years; 10.2 (21.0) days for adolescents aged 13 to 17 years; and 11.5 (38.3) days for young adults aged 18 to 24 years.

**Table 2.** Health System Utilization in the Year Before and After Initial Health Record Diagnosis of Somatic Symptom and Related Disorders by Location of Initial Diagnosis, Overall Cohort^a^

Health system utilization	Outpatient (n = 17 893)			Emergency department (n = 13 310)			Inpatient (n = 2069)		
	1 y Before diagnosis	1 y After diagnosis	*P* value	1 y Before diagnosis	1 y After diagnosis	*P* value	1 y Before diagnosis	1 y After diagnosis	*P* value
**Acute care use**									
Mental health–related visits									
Emergency department	792 (4.4)	748 (4.2)	.18	1451 (10.9)	1667 (12.5)	<.001	330 (15.9)	362 (17.5)	.10
Inpatient	341 (1.9)	415 (2.3)	.001	476 (3.6)	740 (5.6)	<.001	249 (12.0)	327 (15.8)	<.001
All-cause visits									
Emergency department	5749 (32.1)	5586 (31.2)	.03	9090 (68.3)	8673 (65.2)	<.001	1600 (77.3)	1341 (64.8)	<.001
Median (IQR)	0 (0-1)	0 (0-1)	.005	0 (0-3)	0 (0-3)	.11	2 (1-4)	1 (0-3)	<.001
Inpatient	1015 (5.7)	1007 (5.6)	.83	1512 (11.4)	1950 (14.7)	<.001	799 (38.6)	779 (37.7)	.46
**Ambulatory visits**									
Mental health–related visits									
Family physician	4771 (26.7)	6401 (35.8)	<.001	3999 (30.0)	4477 (33.6)	<.001	776 (37.5)	882 (42.6)	<.001
Median (IQR)	0 (0-1)	0 (0-1)	<.001	0 (0-1)	0 (0-1)	<.001	0 (0-1)	0 (0-2)	.02
Psychiatrist	1501 (8.4)	2051 (11.5)	<.001	1479 (11.1)	2013 (15.1)	<.001	708 (34.2)	832 (40.2)	<.001
Median (IQR)	0 (0-0)	0 (0-0)	<.001	0 (0-0)	0 (0-0)	<.001	0 (0-1)	0 (0-2)	<.001
Pediatrician	623 (3.5)	707 (4.0)	.002	600 (4.5)	650 (4.9)	.04	242 (11.7)	287 (13.9)	.004
Median (IQR)	0 (0-0)	0 (0-0)	.16	0 (0-0)	0 (0-0)	.006	0 (0-0)	0 (0-0)	.73
Any ambulatory	5601 (31.3)	7425 (41.5)	<.001	4755 (35.7)	5439 (40.9)	<.001	1167 (56.4)	1339 (64.7)	<.001
Median (IQR)	0 (0-1)	0 (0-2)	<.001	0 (0-1)	0 (0-2)	<.001	1 (0-4)	2 (0-6)	<.001
All-cause outpatient visits									
Family physician	15 543 (86.9)	15 487 (86.6)	.32	11 368 (85.4)	11 484 (86.3)	.01	1804 (87.2)	1763 (85.2)	.02
Median (IQR)	3 (1-6)	3 (1-6)	<.001	3 (1-7)	4 (1-7)	<.001	4 (2-8)	4 (1-8)	.60
Pediatrician	2011 (11.2)	1878 (10.5)	.001	1991 (15.0)	2183 (16.4)	<.001	766 (37.0)	679 (32.8)	<.001
Median (IQR)	0 (0-0)	0 (0-0)	.14	0 (0-0)	0 (0-0)	<.001	0 (0-2)	0 (0-1)	.08
Any ambulatory	15 936 (89.1)	15 883 (88.8)	.32	11 775 (88.5)	11 945 (89.7)	<.001	1971 (95.3)	1955 (94.5)	.21
Median (IQR)	3 (1-7)	4 (2-7)	<.001	4 (2-8)	4 (2-9)	<.001	7 (3-13)	7 (3-13)	<.001

Abbreviation: IQR, interquartile range.

^a^ Data are presented as No. (%) unless otherwise specified.

**Table 3.** Health System Utilization in the Year Before and After Initial Health Record Diagnosis of Somatic Symptom and Related Disorders by Location of Initial Diagnosis, Age-Stratified

Health system utilization	Outpatient^a^			Emergency department^b^			Inpatient^c^		
	1 y Before diagnosis	1 y After diagnosis	*P* value	1 y Before diagnosis	1 y After diagnosis	*P* value	1 y Before diagnosis	1 y After diagnosis	*P* value
**Acute care use**									
Mental health–related visits									
Emergency department, No. (%)									
4-12 y	28 (1.4)	19 (1.0)	.18	38 (2.4)	69 (4.3)	<.001	17 (5.5)	30 (9.7)	.03
13-17 y	177 (5.2)	187 (5.5)	.55	354 (11.3)	436 (13.9)	<.001	120 (16.1)	135 (18.1)	.23
18-24 y	587 (4.7)	542 (4.3)	.10	1059 (12.4)	1162 (13.6)	.004	193 (19.0)	197 (19.4)	.77
Inpatient, No. (%)									
4-12 y	10 (0.5)	24 (1.2)	.01	12 (0.7)	27 (1.7)	.005	NA	22 (7.1)	<.001
13-17 y	109 (3.2)	147 (4.3)	.004	142 (4.5)	223 (7.1)	<.001	94 (12.6)	138 (18.5)	<.001
18-24 y	222 (1.8)	244 (1.9)	.23	322 (3.8)	490 (5.7)	<.001	151 (14.9)	167 (16.4)	.21
All-cause visits									
Emergency department, No. (%)									
4-12 y	563 (28.8)	526 (26.9)	.13	951 (59.1)	828 (51.5)	<.001	248 (80.3)	175 (56.6)	<.001
13-17 y	1151 (34.0)	1129 (33.3)	.50	2130 (67.8)	2052 (65.3)	.01	554 (74.5)	469 (63.0)	<.001
18-24 y	4035 (32.2)	3931 (31.3)	.10	6009 (70.2)	5793 (67.7)	<.001	798 (78.5)	697 (68.6)	<.001
Emergency department, median (IQR)									
4-12 y	0 (0-1)	0 (0-1)	<.001	1 (0-2)	1 (0-2)	<.001	2 (1-4)	1 (0-2)	<.001
13-17 y	0 (0-1)	0 (0-1)	.21	1 (0-3)	1 (0-3)	.04	2 (0-4)	1 (0-3)	.02
18-24 y	0 (0-1)	0 (0-1)	.15	1 (0-3)	1 (0-3)	.06	2 (1-4)	1 (0-4)	.07
Inpatient, No. (%)									
4-12 y	112 (5.7)	120 (6.1)	.47	107 (6.7)	141 (8.8)	.01	108 (35.0)	98 (31.7)	.29
13-17 y	261 (7.7)	304 (9.0)	.02	378 (12.0)	515 (16.4)	<.001	294 (39.5)	294 (39.5)	>.99
18-24 y	642 (5.1)	583 (4.6)	.05	1027 (12.0)	1294 (15.1)	<.001	397 (39.1)	387 (38.1)	.59
**Ambulatory visits**									
Mental health–related visits									
Family physician, No. (%)									
4-12 y	279 (14.2)	397 (20.3)	<.001	198 (12.3)	233 (14.5)	.04	42 (13.6)	60 (19.4)	.02
13-17 y	742 (21.9)	1059 (31.3)	<.001	836 (26.6)	960 (30.6)	<.001	257 (34.5)	303 (40.7)	.002
18-24 y	3750 (29.9)	4945 (39.4)	<.001	2965 (34.6)	3284 (38.4)	<.001	477 (46.9)	519 (51.1)	.02
Psychiatrist, No. (%)									
4-12 y	110 (5.6)	168 (8.6)	<.001	81 (5.0)	148 (9.2)	<.001	69 (22.3)	99 (32.0)	<.001
13-17 y	372 (11.0)	550 (16.2)	<.001	395 (12.6)	589 (18.7)	<.001	311 (41.8)	349 (46.9)	.01
18-24 y	1019 (8.1)	1333 (10.6)	<.001	1003 (11.7)	1276 (14.9)	<.001	328 (32.3)	384 (37.8)	<.001
Pediatrician, No. (%)									
4-12 y	208 (10.6)	312 (15.9)	<.001	208 (12.9)	254 (15.8)	.002	57 (18.4)	73 (23.6)	.05
13-17 y	276 (8.1)	311 (9.2)	.04	306 (9.7)	343 (10.9)	.04	166 (22.3)	208 (28.0)	.001
18-24 y	139 (1.1)	84 (0.7)	<.001	86 (1.0)	53 (0.6)	<.001	19 (1.9)	6 (0.6)	.001
Any ambulatory, No. (%)									
4-12 y	456 (23.3)	674 (34.4)	<.001	369 (22.9)	476 (29.6)	<.001	122 (39.5)	155 (50.2)	.001
13-17 y	1037 (30.6)	1413 (41.7)	<.001	1122 (35.7)	1324 (42.1)	<.001	460 (61.8)	533 (71.6)	<.001
18-24 y	4108 (32.7)	5338 (42.5)	<.001	3264 (38.1)	3639 (42.5)	<.001	585 (57.6)	651 (64.1)	<.001
Any ambulatory, median (IQR)									
4-12 y	0 (0-0)	0 (0-1)	<.001	0 (0-0)	0 (0-1)	<.001	0 (0-1)	1 (0-3)	<.001
13-17 y	0 (0-1)	0 (0-2)	<.001	0 (0-1)	0 (0-2)	<.001	1 (0-4)	2 (0-7)	<.001
18-24 y	0 (0-1)	0 (0-2)	<.001	0 (0-1)	0 (0-2)	<.001	1 (0-5)	2 (0-6)	<.001
**All-cause outpatient visits**									
Family physician, No. (%)									
4-12 y	1632 (83.4)	1627 (83.1)	.80	1264 (78.6)	1233 (76.7)	.10	245 (79.3)	223 (72.2)	.01
13-17 y	2882 (85.1)	2910 (85.9)	.27	2631 (83.7)	2675 (85.1)	.06	623 (83.7)	625 (84.0)	.86
18-24 y	11 029 (87.9)	10 950 (87.3)	.09	7473 (87.3)	7576 (88.5)	.003	936 (92.1)	915 (90.1)	.05
Pediatrician, No. (%)									
4-12 y	661 (33.8)	773 (39.5)	<.001	693 (43.1)	828 (51.5)	<.001	237 (76.7)	225 (72.8)	.17
13-17 y	775 (22.9)	721 (21.3)	.02	903 (28.7)	1048 (33.4)	<.001	448 (60.2)	415 (55.8)	.02
18-24 y	575 (4.6)	384 (3.1)	<.001	395 (4.6)	307 (3.6)	<.001	81 (8.0)	39 (3.8)	<.001
Any ambulatory, No. (%)									
4-12 y	1755 (89.6)	1768 (90.3)	.43	1419 (88.2)	1429 (88.9)	.51	295 (95.5)	294 (95.1)	.84
13-17 y	3025 (89.3)	3047 (90.0)	.33	2801 (89.1)	2868 (91.3)	.001	716 (96.2)	713 (95.8)	.68
18-24 y	11 156 (88.9)	11 068 (88.2)	.05	7555 (88.3)	7648 (89.3)	.006	960 (94.5)	948 (93.3)	.19
Any ambulatory, median (IQR)									
4-12 y	4 (2-7)	4 (2-7)	.16	3 (2-7)	4 (2-7)	<.001	7 (3-12)	7 (3-12)	.92
13-17 y	3 (1-6)	4 (2-7)	<.001	4 (2-7)	4 (2-8)	<.001	7 (4-13)	8 (4-14)	<.001
18-24 y	3 (1-7)	3 (1-7)	<.001	4 (2-9)	5 (2-9)	<.001	7 (3-13)	7 (3-13)	.02

Abbreviations: IQR, interquartile range; NA, not applicable.

^a^ 1958 patients aged 4 to 12 years; 3387 aged 13 to 17 years; and 12 548 aged 18 to 24 years.

^b^ 1608 patients aged 4 to 12 years; 3142 aged 13 to 17 years; and 8560 aged 18 to 24 years.

^c^ 309 patients aged 4 to 12 years; 774 aged 13 to 17 years; and 1016 aged 18 to 24 years.

Children, adolescents, and young adults first diagnosed with an SSRD in an ED setting had a mean (SD) of 2.5 (4.3) ED visits (median [IQR], 0 [0-3]; *P* < .001) leading up to their diagnosis. ED visits did not change in the year after diagnosis, and more patients were hospitalized in the year after their diagnosis than in the year before it (1950 of 13 310 [14.7%] vs 1512 of 13 310 [11.4%], respectively; *P* = <.001).

Among those diagnosed in an outpatient setting, there was no clinically significant change in the proportion who visited the ED for mental health concerns or for any reason in the year before and after their index visit. Compared with the year before diagnosis (341 of 17 893 [1.9%]), more patients were hospitalized in the year after diagnosis (415 of 17 893 [2.3%]) for mental health concerns (*P* = .001), whereas the proportion with hospitalizations for any cause was unchanged.

#### Ambulatory Care Before and After Diagnosis

Among children, adolescents, and young adults hospitalized at their index diagnosis, the mean (SD) number of ambulatory visits in the year before and after diagnosis was 10.0 (15.2) (median [IQR], 7 [3-13]) and 10.8 (14.7) (median [IQR], 7 [3-13]; *P* < .001) (Table 2). Before the index visit, among the 2069 hospitalized patients, 776 (37.5%) had seen a family physician, 708 (34.2%) had seen a psychiatrist, and 242 (11.7%) had seen a pediatrician for a mental health concern, proportions that rose only marginally in the year after diagnosis (to 882 patients [42.6%] for family physician visits [*P* < .001], 832 [40.2%] for psychiatrist visits [*P* < .001], and 287 [13.9%] for pediatrician visits [*P* = .004]). In the year after diagnosis for hospitalized patients, 730 of 2069 (35.3%) children, adolescents, and young adults did not have any ambulatory care visits to physicians for mental health concerns; 7866 of 13 310 (59.1%) did not receive mental health care in an emergency department setting; and 10 467 of 17 893 (58.5%) did not receive mental health care in an outpatient setting (*P* < .001). Among hospitalized individuals, school-aged children (4-12 years) had particularly poor physician follow-up for mental health, with 154 of 309 (48.8%) having no mental health visits within a year of their index hospitalization (Table 3).

Among those diagnosed in the ED, only a small increase in the proportion of individuals with ambulatory mental health visits was observed after diagnosis (4755 of 13 310 [35.7%] before vs 5439 of 13 310 [40.9%] after; *P* < .001). Of those diagnosed in an ambulatory setting, 7425 of 17 893 (41.5%) had a mental health–related outpatient visit in the year after diagnosis vs 5601 of 17 893 (31.3% before diagnosis (*P* < .001) (Table 2).

### Health Care Costs

The estimated total 2-year expenditure of the cohort of 33 272 children, adolescents, and young adults with an SSRD was $327 562 849 (all values in Canadian dollars; exchange rate, CAD$1 = US$0.74), with a mean (SD) of $9845 ($39 725) and a median (IQR) of $2401 ($960-$7019) per patient. Among those diagnosed during a hospitalization, individuals had a 2-year mean (SD) and median (IQR) per-patient expenditure of $51 424 ($100 416) and $21 997 ($12 510-$45 841), respectively. Hospital readmission was the largest contributor to these costs after the index year. Ambulatory care accounted for a relatively small proportion of total costs (Figure). Two-year mean (SD) per-patient expenditure was $9562 ($24 959) and $5249 ($32 928) for patients diagnosed in ED and outpatient settings, respectively. Costs before and after diagnosis overall and by sector are shown in eTable 4 in the Supplement.

**Figure.**
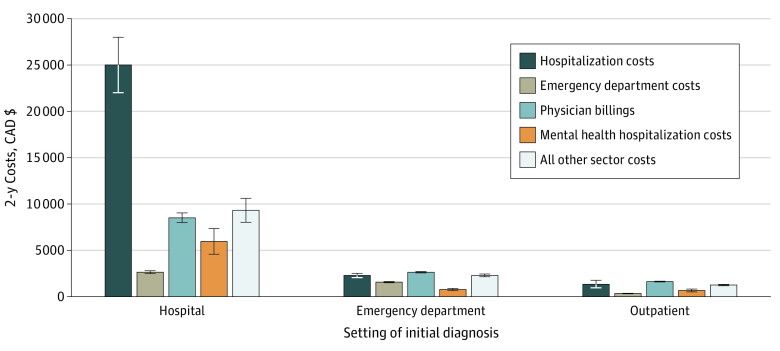
Health System Cost in the Year Before and After Diagnosis Categorized by Setting of Initial Diagnosis of Somatic Symptom and Related Disorders Graph shows mean 2-year costs; error bars represent 95% CIs. CAD$ indicates Canadian dollars.

## Discussion

In this population-based cohort study, we identified and described a population of children, adolescents, and young adults with health system records of an SSRD who have substantial health system use and costs. Per capita costs of hospitalized young people with this disorder were 16 to 20 times the published costs of their peers in Ontario, which range from $1494 to $1843 among young people aged 5 to 24 years.^26^ We report frequent health care use in the year leading up to a health record diagnosis that continues in the year after diagnosis, and our results suggest that follow-up care by physicians and specialists for mental health care was poor. The frequent health care use before index diagnosis suggests that SSRDs may go under-recognized for a prolonged period. The low rate of physician follow-up for mental health care and high rates of ongoing non–mental health system use suggest that a diagnosis in and of itself is not sufficient to lead to a shift in the treatment trajectory toward what might potentially be more effective care for the presenting signs and symptoms. Importantly, despite having a primary mental health condition, many patients did not receive timely mental health support from a physician, particularly hospitalized school-aged children. Initiatives to recognize SSRDs and to ensure supports are put in place early are warranted.

Somatic symptoms are a common reason for medical visits in young children.^1,2,4,5^ In adults, prevalence estimates for those with any SSRD are upwards of one-third of patients in primary care, and approximately 8% of patients present with multiple concurrent somatic symptoms.^14,17,23^

Studies of adults with an SSRD have reported a mean of 12.8 outpatient physician or nurse practitioner visits per year with nonspecific symptoms,^23^ and there is high comorbidity with other mental illnesses, including depression, anxiety, and substance use disorder.^27,28,29^ Our cohort of children, adolescents, and young adults similarly had high numbers of outpatient visits, inpatient hospitalizations, and emergency visits for comorbid mental illness. A large proportion of revisits in the year after diagnosis in our cohort were for mental illness, yet outpatient physician visits for mental health concerns remained low. Early mental health consultation during inpatient hospitalization has been shown to reduce the length of hospital stay in children with comorbid mental health diagnoses, including those with SSRDs.^30^ A number of feasible and some cost-effective primary care and transitional interventions have been identified to potentially reduce psychiatric readmission or improve quality of life and could be considered in the population.^31,32,33,34^

Health care costs of children, adolescents, and young adults in Canada with SSRDs have not, to our knowledge, been previously reported. Our data suggest that per capita costs for these patients are 6 to 8 times the costs for age-related peers in Ontario.^26^ Among hospitalized children, adolescents, and young adults specifically, health system costs were even higher and were on par with costs among those hospitalized with chronic complex conditions with technology dependence (ie, gastrostomy tubes, tracheostomies, ventriculoperitoneal shunts, etc). Hospitalized patients with these chronic conditions have received much attention in recent years.^26,35^ Yet, unlike their peers with complex medical conditions, these children, adolescents, and young adults have not been subject to the same health system responsiveness for care navigation, coordination, and interventions.^35,36,37,38,39^ Costs related to SSRDs are high in hospitalized adults in Italy, with mean annual per-patient costs of €47 540.^9^ In both inpatients and outpatients in Holland, mean direct and indirect costs were estimated at €6815 per patient per year.^40^ Others have reported $6354 (US dollars) per-patient annual health care expenditure.^41^

Identifying those who frequently use the health system has been recognized as an important step to improve health system sustainability, quality of care, and patient outcomes.^35,42^ This identification facilitates improved patient management through better understanding of the clinical needs of the population of patients who frequently use health services and allows services to be targeted appropriately.^35^ Care for those with an SSRD has been described as fragmented, uncoordinated, and difficult to navigate because of the multiple health care practitioners involved.^1,2,43,44^ Thus, this is a patient population in which high-intensity, coordinated, and timely integrated care may be of benefit. Integrated models of care for children, adolescents, and young adults with an SSRD have shown some promise in improving outcomes through a coordinated approach that de-emphasizes medicalizing symptoms, and instead supports the mind-body connection in outpatient settings.^2,45,46,47^ Because such care models can be costly, they need to be targeted to those who would benefit most or to those who incur the greatest cost to maximize the potential for cost offset. Importantly, in pediatric populations, early-life health care use for functional disorders is associated with future health care use. Other researchers have also shown that SSRDs in adolescence are predictive of severe mental illness in adults, as measured by hospital-based mental health care, even when controlling for confounders.^48^ Although two-thirds of our cohort were emerging adults, a sizable proportion were school-aged children and adolescents. Interventions in early childhood have the potential to both reduce the distress of this patient population and reduce costs associated with frequent health care use in this patient population.

### Limitations

There are important limitations to this study. We ascertained our cohort from individuals who had a health record rather than a clinical diagnosis of an SSRD and, therefore, did not capture individuals who had functional symptoms but were not recorded in the inpatient and ED discharge records or in physician billing records. Therefore, we likely underestimated the overall health system use and costs of SSRDs. We do not have health and administrative data on social workers and psychologists who may provide some mental health treatment for individuals with functional disorders; therefore, we cannot fully describe the mental health services delivered to these children before and after diagnosis. Databases do not capture private drug and home care (eg, occupational therapy and physiotherapy) coverage and care provided in community health centers (<1% of the population),^49^ which do not bill through fee-for-service. Importantly, we do not capture indirect health costs, including caregiver costs, particularly from lost parental employment time. There is a lack of a unified and validated definition of SSRD using administrative data and for other research purposes. Diagnoses were from physicians and noted in health records rather than obtained through any specific SSRD measurement tool for diagnosis, as no such validated tool currently exists or is available in administrative data.

Despite these limitations, our findings suggest an ability to identify a group of children, adolescents, and young adults using administrative data who frequently use the health care system and who may not be receiving mental health care from physicians before and after their mental health diagnosis, despite their frequent health system contact. Our findings may be applicable to other high-income countries that have reported high costs for health system use for adults with SSRDs. Our study suggests that there may be ongoing barriers after diagnosis to receiving needed care and, further, that persistence of frequent use of non–mental health services may be contributing to suboptimal use of the health care system. We have identified a population to target for earlier disease recognition and to streamline pathways for care delivery.

## Conclusions

SSRDs are common among children, adolescents, and young adults. This cohort study found that pediatric populations in Ontario had frequent health system use and costs leading up to their diagnosis that persisted after diagnosis. Only a small proportion received physician-delivered mental health care despite having a mental health diagnosis, suggesting that there is opportunity to have care delivery better aligned with patients’ needs upon diagnosis. Such alignment may help to address their mental health concerns, lead to better patient outcomes, and reduce inappropriate care and associated costs.

## Notes

Supplement. **eTable 1.** Data Sources **eTable 2.** International Classification of Diseases Ninth and Tenth Revision-Canada Edition Codes Used for Case Finding of a Health Administrative Record of Somatic Symptom and Related Disorders **eTable 3.** Primary Discharge Diagnoses at Index Visit **eTable 4.** Health System Costs in the Year Before and the Year After Initial Health Record Diagnosis of Somatic Symptom and Related Disorders by Location of Initial Diagnosis, All Ages
