# Supplementary material for: Health Care Use and Costs of Children, Adolescents, and Young Adults With Somatic Symptom and Related Disorders
Source: JAMA Netw Open. 2020 Jul 23;3(7):e2011295. doi: 10.1001/jamanetworkopen.2020.11295 (PMC7378752; doi:10.1001/jamanetworkopen.2020.11295)
Supplement: Supplement. — eTable 1. Data Sources eTable 2. International Classification of Diseases Ninth and Tenth Revision-Canada Edition Codes Used for Case Finding of a Health Administrative Record of Somatic Symptom and Related Disorders eTable 3. Primary Discharge Diagnoses at Index Visit eTable 4. Health System Costs in the Year Before and the Year After Initial Health Record Diagnosis of Somatic Symptom and Related Disorders by Location of Initial Diagnosis, All Ages [file jamanetwopen-3-e2011295-s001.pdf]

Supplementary Online Content

Saunders NR, Gandhi S, Chen S, et al. Health care use and costs of children, adolescents, and young adults with somatic symptom and related disorders. *JAMA Netw Open*. 2020;3(7):e2011295. doi:10.1001/jamanetworkopen.2020.11295

eTable 1. Data Sources

eTable 2. International Classification of Diseases Ninth and Tenth Revision-Canada Edition Codes Used for Case Finding of a Health Administrative Record of Somatic Symptom and Related Disorders[a]

eTable 3. Primary Discharge Diagnoses[a] at Index Visit

eTable 4. Health System Costs in the Year Before and the Year After Initial Health Record Diagnosis of Somatic Symptom and Related Disorders by Location of Initial Diagnosis, All Ages

This supplementary material has been provided by the authors to give readers additional information about their work.

© 2020 Saunders NR et al. *JAMA Network Open.*

**eTable 1.** Data Sources		
**Data source**	**Description**	**Measures**
Registered Persons Database (RPDB)	Provides basic demographic information about anyone who has ever received an Ontario health card number.	Age<br>Sex<br>OHIP eligibility<br>Income quintile<br>Rurality
Immigration, Refugees and Citizenship Canada (IRCC)'s Permanent Resident Database	Includes immigration application records for people who initially applied to land in Ontario, since 1985. Contains permanent residents' demographic information such as country of citizenship, level of education, mother tongue, and landing date.	Immigrant category
Ontario Health Insurance Plan (OHIP)	Contains claims data paid by OHIP for services provided by all eligible health care providers including, physicians, groups, and laboratories	Ambulatory (outpatient) visits<br>Physician specialty<br>Physician claims<br>OHIP diagnosis code<br>Healthcare costs
Discharge Abstract Database (DAD)	This database contains information about patient separations, such as clinical data (diagnoses, procedures) and resource consumption, defined using case mixed group and resource intensity weight	Acute hospitalization admissions<br>Discharge diagnosis<br>Healthcare costs
National Ambulatory Care Reporting System (NACRS)	Captures data on hospital and community based ambulatory care, notably, day surgery, outpatient clinics, and emergency departments.	Emergency department visits<br>Discharge diagnosis<br>Healthcare costs
Ontario Mental Health Reporting System (OMHRS)	Collects data on patients in adult designated inpatient mental health beds in acute and psychiatric care facilities. Capture admission and discharge dates, diagnostic information, and service use.	Inpatient psychiatric admissions<br>Discharge diagnosis<br>Healthcare costs
Client Agency Program Enrolment (CAPE)	Dataset indicates the enrolment of patients in a primary care model, enrolment status, and program type.	Primary care program enrolment<br>Usual provider of primary care assignment<br>Healthcare costs
Corporate Provider Database (CPDB)	Contains information about all physician and other providers (such as chiropractors, physiotherapists, and optometrist). Data elements include demographic characteristics, eligibility, specialty, and practice location.	Usual provider of primary care specialty
Ontario Drug Benefit (ODB)	Contains claims for prescription drugs covered the Ontario Drug Benefit Program.	Healthcare costs
National Rehabilitation Reporting System (NRS)	Contains information from inpatient rehabilitation facilities, including referral, admission and discharge records; and health and functional characteristics	Healthcare costs
Continuing Care Reporting System (CCRS)	Contains information on residents receiving facility-based continuing care services, including hospital-based continuing care, and residential care (long-term care).	Healthcare costs
Home care database (HCD)	Includes data on services provided through the Ontario Community Care Access Centres, including assessment and intake data, services, admission and discharge records.	Healthcare costs
Assisted Devices Program (ADP)	Contains data on Ontario residents who have long-term physical disabilities, and receive personalized assistive devices such as insulin pumps and supplies, and other equipment.	Healthcare costs

© 2020 Saunders NR et al. *JAMA Network Open.*

eTable 2. International Classification of Diseases Ninth and Tenth Revision-Canada Edition Codes Used for Case Finding of a Health Administrative Record of Somatic Symptom and Related Disorders[a]

DIAGNOSIS	ICD-10-CA	ICD9	ICD-9 and 10 DESCRIPTIONs	DSM-IV DESCRIPTION
Somatization disorder	F45	300.81	Somatization disorder	Somatization disorder
		300.82		Somatoform disorder - undifferentiated and NOS
		300.7	Hypochondriasis	
		306.4	Psychogenic gastrointestinal disease	
		307.89	Psychogenic pain NEC	Pain disorder associated with both psychological factors and a general medical condition
		306.8	Psychogenic disorder NEC	
		306.9	Psychogenic disorder NOS	
Conversion disorder	F44	300.12	Psychogenic amnesia	Dissociative amnesia
		300.13	Psychogenic fugue	Dissociative fugue
		300.10	Hysteria NOS	
		300.11	Conversion disorder	Conversion disorder
		300.14	Multiple personality	Dissociative identity disorder
		300.15	Dissociative react NOS	Dissociative disorder NOS
Psychological and behavioural factors associated with disorders or diseases classified elsewhere	F54	316	Psychic factors associated with diseases classified elsewhere	
Unspecified behavioural syndromes associated with physiological disturbances and physical factors	F59	306.9	Psychogenic disorder NOS	
Factitious disorder	F68	300.16	Factitious ill w symptom	Factitious disorder with predominantly psychological signs and symptoms
		301.51	Chronic factitious illness with physical symptoms	
Irritable bowel syndrome	K58	564.1	Irritable colon	
Fibromyalgia	M79.7	729.1	Myalgia and myositis NOS	
Chronic intractable pain	R52.1	780.9	General symptoms NEC	
Other chronic pain	R52.2	780.9	General symptoms NEC	
Pain, unspecified	R52.9	780.9	General symptoms NEC	
Chronic fatigue syndrome	F48.0	300.5	Neurasthenia	
	R53	780.79		

NOS, Not otherwise specified; NEC, not elsewhere classifiable.

[a] Outpatient billings use only ICD9 codes (first three digits), hospitalization and emergency departments use ICD-10-CA codes in any position (primary diagnosis or any diagnosis contributing to length of stay).

© 2020 Saunders NR et al. *JAMA Network Open.*

eTable 3. Primary Discharge Diagnoses[a] at Index Visit

**Primary discharge diagnoses at index visit.**			
**Index visit in emergency department**		**Index diagnosis during hospitalization**	
**All Ages**			
**Somatization as primary diagnosis**	7,671 (57.6)	**Somatization as primary diagnosis**	777 (37.6)
**Primary diagnoses that were not SSRD**	5,639 (42.4)	**Primary diagnoses that were not SSRD**	1,292 (62.4)
Other and unspecified abdominal pain, R104	1,525 (27.0)	Other and unspecified abdominal pain, R104	67 (5.2)
Gastroenteritis and colitis of unspecified origin, A099	231 (4.1)	Severe depressive episode without psychotic symptoms F322	36 (2.8)
Constipation, K590	211 (3.7)	Adjustment disorders, F432	21 (1.6)
Low back pain, M545	188 (3.3)	Constipation, K590	18 (1.4)
Anxiety disorder, unspecified, F419	176 (3.1)	Depressive episode, unspecified, F329	17 (1.3)
Other	3,308 (58.7)	Other	1,133 (87.7)
**4 to 12 years**			
**Somatization as primary diagnosis**	947 (58.9)	**Somatization as primary diagnosis**	151 (48.9)
**Primary diagnoses that were not SSRD**	661 (41.1)	**Primary diagnoses that were not SSRD**	158 (51.1)
Other and unspecified abdominal pain, R104	229 (34.6)	Other and unspecified abdominal pain, R104	12 (7.6)
Constipation, K590	50 (7.6)	Constipation, K590	7 (4.4)
Gastroenteritis and colitis of unspecified origin, A099	22 (3.3)	Other and unspecified convulsions, R5688	6 (3.8)
Anxiety disorder, unspecified, F419	21 (3.2)	*Collapsed due to small cell sizes	n/a
Headache, R51	18 (2.7)	*Collapsed due to small cell sizes	n/a
Other	321 (48.6)	Other	133 (84.2)
**Age 13 to 17 years**			
**Somatization as primary diagnosis**	1803 (57.4)	**Somatization as primary diagnosis**	286 (38.4)
**Primary diagnoses that were not SSRD**	1,339 (42.6)	**Primary diagnoses that were not SSRD**	458 (61.6)
Other and unspecified abdominal pain, R104	409 (30.5)	Severe depressive episode without psychotic symptoms, F322	32 (7.0)
Anxiety disorder, unspecified, F419	49 (3.7)	Adjustment disorders, F432	19 (4.1)
Constipation, K590	47 (3.5)	Other and unspecified abdominal pain, R104	19 (4.1)
Gastroenteritis and colitis of unspecified origin, A099	40 (3.0)	Depressive episode, unspecified, F329	14 (3.1)
Headache, R51	26 (1.9)	Headache, R51	11 (2.4)
Other	768 (57.4)	Other	363 (79.3)
**Age 18 to 24 years**			
**Somatization as primary diagnosis**	4,921 (57.5)	**Somatization as primary diagnosis**	340 (33.5)
**Primary diagnoses that were not SSRD**	3,639 (42.5)	**Primary diagnoses that were not SSRD**	458 (66.5)
Other and unspecified abdominal pain, R104	887 (24.4)	Other and unspecified abdominal pain, R104	36 (5.3)
Gastroenteritis and colitis of unspecified origin, A099	169 (4.6)	Psychotic disorder NOS, 2989	11 (1.6)
Low back pain, M545	162 (4.5)	Epilepsy, unspecified, not stated as intractable, G4090	10 (1.5)
Constipation, K590	114 (3.1)	Mood Disorder, F39	10 (1.5)
Anxiety disorder, unspecified, F419	106 (2.9)	Gastroenteritis and colitis of unspecified origin, A099	9 (1.3)
Other	2,201 (60.5)	Other	600 (88.8)

© 2020 Saunders NR et al. *JAMA Network Open.*

eTable 4. Health system costs in year prior to and year following initial health record diagnosis of somatic symptom and related disorders by location of initial diagnosis, All ages.

**All Ages**									
	**Diagnostic setting**								
	**First diagnosis of somatization in an outpatient setting**			**First diagnosis of somatization in an emergency department**			**First diagnosis of somatization during a hospitalization**		
	N = 17 893			N = 13 310			N = 2069		
Health System Costs	One year prior to diagnosis	One year following diagnosis	***p***	One year prior to diagnosis	One year following diagnosis	***p***	One year prior to diagnosis	One year following diagnosis	***p***
**OVERALL HEALTH SYSTEM COSTS (CANADIAN DOLLARS)**									
Mean ± SD	2,367.82 ± 10,664.70	2,881.79 ± 28,243.13	0.01	4,325.58 ± 15,618.16	5,236.34 ± 14,565.59	<0.00	31,988.77 ± 72,979.52	19,434.98 ± 43,142.99	<0.001
Median (IQR)	573.43 (269.33-1,434.97)	560.82 (234.65-1,518.40)		1,550.22 (799.81-3,451.59)	1,719.79 (636.55-4,455.38)		14,094.56 (9,009.61-26,654.52)	4,960.31 (1,658.48-16,373.68)	
**HEALTH SYSTEM COSTS BY SECTOR (CANADIAN DOLLARS)**									
**Hospitalizations**									
Mean ± SD	528.43 ± 4,948.25	830.91 ± 25,695.33	0.10	1,010.38 ± 10,356.85	1,273.74 ± 7,496.81	0.01	17,509.97 ± 58,896.48	7,499.04 ± 25,457.08	<0.001
Median (IQR)	-	-		-	-		7,219.62 (3,616.14-13,337.14)	0.00 (0.00-4,818.69)	
**Emergency department visits**									
Mean ± SD	181.02 ± 552.16	168.18 ± 599.84	0.001	900.77 ± 1,319.99	685.92 ± 1,605.42	<0.00	1,603.74 ± 1,864.79	1,030.13 ± 2,026.63	<0.001
Median (IQR)	0.00 (0.00-174.20)	0.00 (0.00-162.57)		553.11 (298.97-1,046.45)	254.04 (0.00-778.71)		1,171.82 (635.91-1,998.46)	411.81 (0.00-1,263.71)	
**Physician billings**									
Mean ± SD	786.67 ± 1,556.25	838.84 ± 2,200.22	0.001	1,173.11 ± 2,086.37	1,457.59 ± 2,788.08	<0.00	4,867.65 ± 6,687.54	3,642.72 ± 8,405.73	<0.001
Median (IQR)	355.97 (186.25-758.62)	342.92 (159.26-788.62)		613.49 (297.92-1,301.67)	772.98 (334.09-1,644.67)		3,063.04 (1,765.80-5,381.66)	1,711.18 (725.47-3,798.28)	
**Mental health hospitalizations**									
Mean ± SD	248.41 ± 4,909.17	412.52 ± 7,195.81	<0.00	279.08 ± 3,854.67	488.86 ± 5,318.77	<0.00	3,810.01 ± 20,991.49	2,146.85 ± 15,358.38	<0.001
Median (IQR)	-	-		-	-		-	-	
**All other sectors**									
Mean ± SD	623.29 ± 4,533.96	631.34 ± 2,560.12	0.80	962.23 ± 4,744.59	1,330.23 ± 4,990.01	<0.00	4,197.41 ± 14,672.20	5,116.25 ± 17,458.49	<0.001
Median (IQR)	100.92 (20.81-377.91)	88.46 (0.00-398.85)		147.03 (27.11-757.02)	360.35 (47.54-1,342.88)		1,177.86 (405.89-3,168.65)	1,345.27 (365.65-3,746.23)	

© 2020 Saunders NR et al. *JAMA Network Open.*
